# Aberrant Neuronal Differentiation and Inhibition of Dendrite Outgrowth Resulting from Endoplasmic Reticulum Stress

**DOI:** 10.1002/jnr.23389

**Published:** 2014-04-10

**Authors:** Koichi Kawada, Takaaki Iekumo, Ryo Saito, Masayuki Kaneko, Seisuke Mimori, Yasuyuki Nomura, Yasunobu Okuma

**Affiliations:** 1 Department of Pharmacology, Chiba Institute of ScienceChiba, Japan; 2 Laboratory of Medical Therapeutics and Molecular Therapeutics, Gifu Pharmaceutical UniversityGifu, Japan; 3 Department of Pharmaceutical Chemistry, Chiba Institute of ScienceChiba, Japan; 4 Department of Pharmacology, Kurume University School of MedicineKurume, Japan

**Keywords:** ubiquitin ligase HRD1, endoplasmic reticulum stress, neuronal differentiation, neurite outgrowth

## Abstract

Neural stem cells (NSCs) play an essential role in development of the central nervous system. Endoplasmic reticulum (ER) stress induces neuronal death. After neuronal death, neurogenesis is generally enhanced to repair the damaged regions. However, it is unclear whether ER stress directly affects neurogenesis-related processes such as neuronal differentiation and dendrite outgrowth. We evaluated whether neuronal differentiation and dendrite outgrowth were regulated by HRD1, a ubiquitin ligase that was induced under mild conditions of tunicamycin-induced ER stress. Neurons were differentiated from mouse embryonic carcinoma P19 cells by using retinoic acid. The differentiated cells were cultured for 8 days with or without tunicamycin and HRD1 knockdown. The ER stressor led to markedly increased levels of ER stress. ER stress increased the expression levels of neuronal marker βIII-tubulin in 8-day-differentiated cells. However, the neurites of dendrite marker microtubule-associated protein-2 (MAP-2)-positive cells appeared to retract in response to ER stress. Moreover, ER stress markedly reduced the dendrite length and MAP-2 expression levels, whereas it did not affect the number of surviving mature neurons. In contrast, HRD1 knockdown abolished the changes in expression of proteins such as βIII-tubulin and MAP-2. These results suggested that ER stress caused aberrant neuronal differentiation from NSCs followed by the inhibition of neurite outgrowth. These events may be mediated by increased HRD1 expression.

New neurons are generated in specific regions of the mammalian brain throughout adult life. Slowly dividing and self-renewing neural stem cells (NSCs) are present in the subventricular zone (SVZ) of the lateral ventricles and in the subgranular zone of the hippocampal dentate gyrus. NSCs generate rapidly proliferating neural progenitor cells that ultimately differentiate to produce thousands of new neurons each day in the adult mammalian brain (Abrous et al., 2005). Neurogenesis declines with age (Molofsky et al., 2006; Pekcec et al., 2008; Knoth et al., 2010) and is impaired by various types of stressors, such as stress hormones (Mirescu and Gould, 2006), and inflammation in regions of the brain (Monje et al., 2003). Natriuretic peptides hormones, which stimulate branch formation and induce axon outgrowth, have wide influence on the development and function of the nervous system in the embryonic and adult mammal (Zhao and Ma, 2009). Differentiation to neuron from proliferative radial glia may underlie the radial organization of neocortex in embryonic development (Noctor et al., 2001). Thus, the regulation of neuronal differentiation and neurite outgrowth plays a pivotal role in adult brain homeostasis and embryonic development.

The endoplasmic reticulum (ER) plays important roles in the processing, glycosylation, and disulfide-bond formation in newly synthesized membranes and secretory proteins. A variety of stressors affect ER function and result in an accumulation of unfolded proteins in the ER lumen. Under such conditions, known as *ER stress*, the unfolded protein response, which consists of translational arrest, ER-chaperone induction, and ER-associated degradation (ERAD), is activated to unload the accumulated nonfunctional proteins (Harding et al., 2002; Schröder and Kaufman, 2005; Ron and Walter, 2007). HRD1 is an E3 ubiquitin ligase that is localized in the ER, expressed during ER stress, and protects against ER-stress-induced apoptosis (Kaneko et al., 2002; Carvalho et al., 2006). HRD1 is a central member of the ubiquitin ligase complexes that are responsible for the recognition and ubiquitination of misfolded proteins in the ER for subsequent degradation by proteasomes (Gauss et al., 2006). HRD1 is deeply involved in the degradation of unfolded amyloid precursor protein through ERAD (Kaneko et al., 2010). Furthermore, we previously demonstrated that HRD1 promotes ubiquitination and degradation of Parkin-associated endothelin receptor-like receptor (Pael-R), a substrate for Parkin, and suppresses Pael-R-induced ER stress and apoptosis (Omura et al., 2006). Parkin is a component of a ubiquitin ligase complex and is encoded by the *PARK2* gene. Mutations in this gene are known to cause a familial form of Parkinson's disease known as *autosomal recessive juvenile Parkinson's disease*.

NSCs of the mammalian brain express nestin; these neurons exhibit self-renewal and pluripotency (Rietze et al., 2001). In a previous study, we demonstrated that neurons and NSCs, but not astrocytes, are colocalized with HRD1 in many regions of the adult brain, such as the hippocampus and SVZ (Omura et al., 2008; Kawada et al., 2011). Therefore, we hypothesized that ER stress responses may be involved in neurogenesis, including proliferation, neuronal differentiation, and neurite outgrowth. In fact, brain ischemia triggers excess neurogenesis in parallel with the induction of various stresses such as oxidative and ER stress (Arvidsson et al., 2002; Deierborg et al., 2010). In addition, brain ischemia positively induces ER stress and increases HRD1 expression near damaged regions (Qi et al., 2004). However, it is not clear whether ER stress directly induces the neurogenesis. This study investigates the influence of ER stress on neuronal differentiation and neurite outgrowth with mouse embryonal carcinoma P19 cells. Additionally, we investigated whether HRD1 was involved in neuronal differentiation and neurite outgrowth.

## Materials and Methods

### Antibodies and Chemicals

Tunicamycin was purchased from Wako Pure Chemical Industries (Osaka, Japan). 2-Deoxy-D-glucose and rabbit polyclonal antibodies against glial fibrillary acidic protein (GFAP), doublecortin (DCX), and HRD1 (C-terminal) were purchased from Sigma-Aldrich (St. Louis, MO). Mouse monoclonal antibodies against nestin, βIII-tubulin, neuronal nuclei protein (NeuN; A60), and microtubule-associated protein-2 (MAP-2) were purchased from Millipore (Temecula, CA). A mouse monoclonal antibody against KDEL (10C3) was purchased from Stressgen Biotechnologies (Victoria, British Columbia, Canada). A rabbit polyclonal antibody against cleaved Notch1 (Val1744) was purchased from Cell Signaling Technology (Danvers, MA). A goat polyclonal antibody against Notch3 (M-20) and a rabbit polyclonal antibody against GADD 153 (CHOP) were purchased from Santa Cruz Biotechnology (Santa Cruz, CA). Horseradish peroxidase (HRP)-conjugated anti-mouse and anti-rabbit IgG antibodies were purchased from GE Healthcare (Buckinghamshire, United Kingdom). An Alexa Fluor 488-conjugated anti-mouse IgG antibody and an Alexa Fluor 546-conjugated anti-rabbit IgG antibody were purchased from Life Technologies (Grand Island, NY). Western Lightning Chemiluminescence Reagent Plus was obtained from PerkinElmer Life Science (Waltham, MA).

### Cell Culture

**Figure 1:**
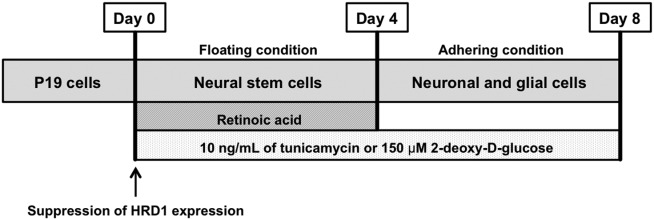
Culture of P19 cells. P19 cells were differentiated into neural stem cells (NSCs) by using 0.5 μM ATRA for 4 days in floating conditions. At 4 days, these cells differentiated into neurons without ATRA for 4 days in adhering conditions. The cells were exposed to tunicamycin from day 0 to day 8. ATRA, all-trans retinoic acid.

Mouse embryonal carcinoma P19 cells were donated by Prof. Yukio Yoneda (Kanazawa University). The culture conditions and time schedules are shown in Figure 1. In brief, the cells were maintained in MEM-α (Life Technologies) containing 10% (v/v) heat-inactivated fetal calf serum (FCS; Life Technologies). The cells were then suspended in MEM-α containing 5% FCS and 0.5 mM all-trans retinoic acid (ATRA; Sigma-Aldrich). These cells were seeded at a density of 1 × 10^5^ cells/ml on 10-cm sterile Petri dishes (AGC Techno Glass, Tokyo, Japan), which were coated with 0.2% agarose gel after counting of viable cell numbers as determined by the trypan blue exclusion test. They were cultured for a period of up to 4 days in vitro in floating conditions. After 4 days, the cells were collected and suspended in MEM-α containing 10% FCS. These cells were seeded at a density of 2 × 10^5^ cells/ml in six-well dishes coated with 75 μg/ml poly-L-lysine. After counting the viable cell numbers determined by the trypan blue exclusion test, the cells were cultured for a period of up to 8 days in vitro under adhering conditions. The cultures were always maintained at 37°C in 95% (v/v) humidified air atmosphere/5% (v/v) CO_2_.

### Immunoblot Analysis

The cell lysates were boiled at 100°C for 10 min in 10 mM Tris-HCl buffer (pH 6.8) containing glycerol, sodium dodecyl sulfate, bromophenol blue, and 2-mercaptoethanol and then stored at −80°C until use. An aliquot (30 μg protein) of the lysates was loaded onto a 6% or 9% polyacrylamide gel for electrophoresis at a constant current of 30 mA for 100 min at room temperature and subsequently blotted onto a polyvinylidene fluoride membrane that was previously treated with 100% methanol. After blocking with 5% skim milk dissolved in 20 mM Tris-HCl buffer (pH 7.5) containing 137 mM NaCl and 0.05% Tween 20 (TBST), the membrane was reacted with primary antibodies against KDEL (1:2,000), GADD 153 (1:1,000), nestin (1:1,000), βIII-tubulin (1:1,000), DCX (1:1,000), MAP-2 (1:1,000), NeuN (1:1,000), GFAP (1:2,000), HRD1 (1:1,000) Notch1 intracellular domain (1:1,000), and GAPDH (1:2,000) for 2 hr at room temperature. After washing three times (5 min each) with TBST, the membranes were incubated with HRP-conjugated secondary antibodies for 1 hr at room temperature. Proteins that were reactive with the antibody were detected by Western Lightning Chemiluminescence Reagent Plus and then quantified with an LAS-3000 luminescent image analyzer (Fujifilm, Tokyo, Japan).

### Semiquantitative RT-PCR Analysis

Total RNA was isolated from the differentiated P19 cells with TRI reagent (Life Technologies) according to the manufacturer's instructions. Total RNA was reverse transcribed to prepare cDNA using a SuperScript VILO cDNA synthesis kit (Life Technologies). An aliquot of cDNA was then amplified by RT-PCR by using 0.8 μM of each primer set for the genes in 20 μl of reaction mixture with GoTaq Hot Start Green Master Mix (Promega, Madison, WI), with denaturation at 95°C for 30 sec, elongation at 72°C for 30 sec, and annealing as per the conditions listed in TableI. The nucleotide sequence of the primers is also denoted in TableI. Reactions were conducted for a suitable number (25–30) of cycles with a 2720 Thermal Cycler (Life Technologies). After the last cycle, a final extension step was performed at 72°C for 10 min, and the PCR products were then analyzed by conducting electrophoresis with a 4% acrylamide gel. These experiments were performed with the same amount of cDNA.

**Table I:** Primers and Conditions Used for RT-PCR Analyses

Gene	Upstream (5′-3′)	Downstream (5′-3′)	Product (bp)	Annealing temperature (°C)
*Hes1*	GCCAATTTGCCTTTCTCATC	AGGCGCAATCCAATATGAAC	384	61
*Hes5*	ATGCTCAGTCCCAAGGAGAA	CGCTGGAAGTGGTAAAGCAG	335	61
*Stat3*	GACCCGCCAACAAATTAAGA	TCGTGGTAAACTGGACACCA	215	61
*Pax6*	AACAACCTGCCTATGCAACC	ACTTGGACGGGAACTGACAC	206	61
*Math1*	GCTTCCTCTGGGGGTTACTC	ACAACGATCACCACAGACCA	341	63
*Math3*	TCTTCGACTGGCAAGGAACT	ACTAATGCTCAGGGGTGGTG	394	63
*NeuroD*	TTGAAGCCATGAATGCAGAG	CTGCTCAGGCAAGAAAGTCC	470	61
*β-Actin*	CCCAGAGCAAGAGAGGTATC	AGAGCATAGCCCTCGTAGAT	340	54.5

### Real-Time RT-PCR Analysis

mRNA expression was measured with the TaqMan-based real-time PCR assay using a 7500 Real-Time PCR system (Life Technologies). An aliquot of cDNA was amplified with 0.4 μM of each primer set for the genes in 20 μl of reaction mixture containing 0.2 mM of each dNTP, 0.625 units Taq DNA polymerase, 10 mM Tris-HCl (pH 8.3), 50 mM KCl, 1.5 mM MgCl_2_, and 0.001% gelatin, with denaturation at 94°C for 1 min, annealing under the conditions listed in TableI, and elongation at 72°C for 1 min.

### Immunocytochemistry

Cells were fixed with cold methanol for 15 min at 4°C and then blocked with Image-iT FX signal enhancer (Life Technologies) for 60 min. Subsequently, the cells were reacted with adequately diluted primary antibodies against MAP-2 (1:200), βIII-tubulin (1:200), nestin (1:200), and HRD1 (1:200) overnight at 4°C. Then the cells were reacted with the corresponding secondary antibody, which was an anti-mouse IgG antibody conjugated with Alexa Fluor 488 (1:200) or an anti-rabbit IgG antibody conjugated with Alexa Fluor 546 (1:200). They were then washed with 0.03% TBST at room temperature. After they had been rinsed for 5 min with TBST, the cells were examined under an LSM 510 Meta confocal microscope (Carl Zeiss, Oberkochen, Germany). The number of βIII-tubulin-positive cells was counted in five different visual fields randomly selected on each coverslip. The number of the positive cells was determined as the sum of those found in the visual fields.

### Measurement of Dendrite Outgrowth

P19 cells were immunostained by MAP-2 antibody as described above. Subsequently, MAP-2-positive cells were analyzed for dendrite length. Average dendrite length was determined by measuring the longest dendrite of about five cells per field/well randomly.

### Statistical Analysis

All data are expressed as mean ± SEM. Statistical significance was determined by *t*-tests or Bonferroni/Dunn tests.

## Results

### Low Concentrations of Tunicamycin Induced ER Stress

**Figure 2:**
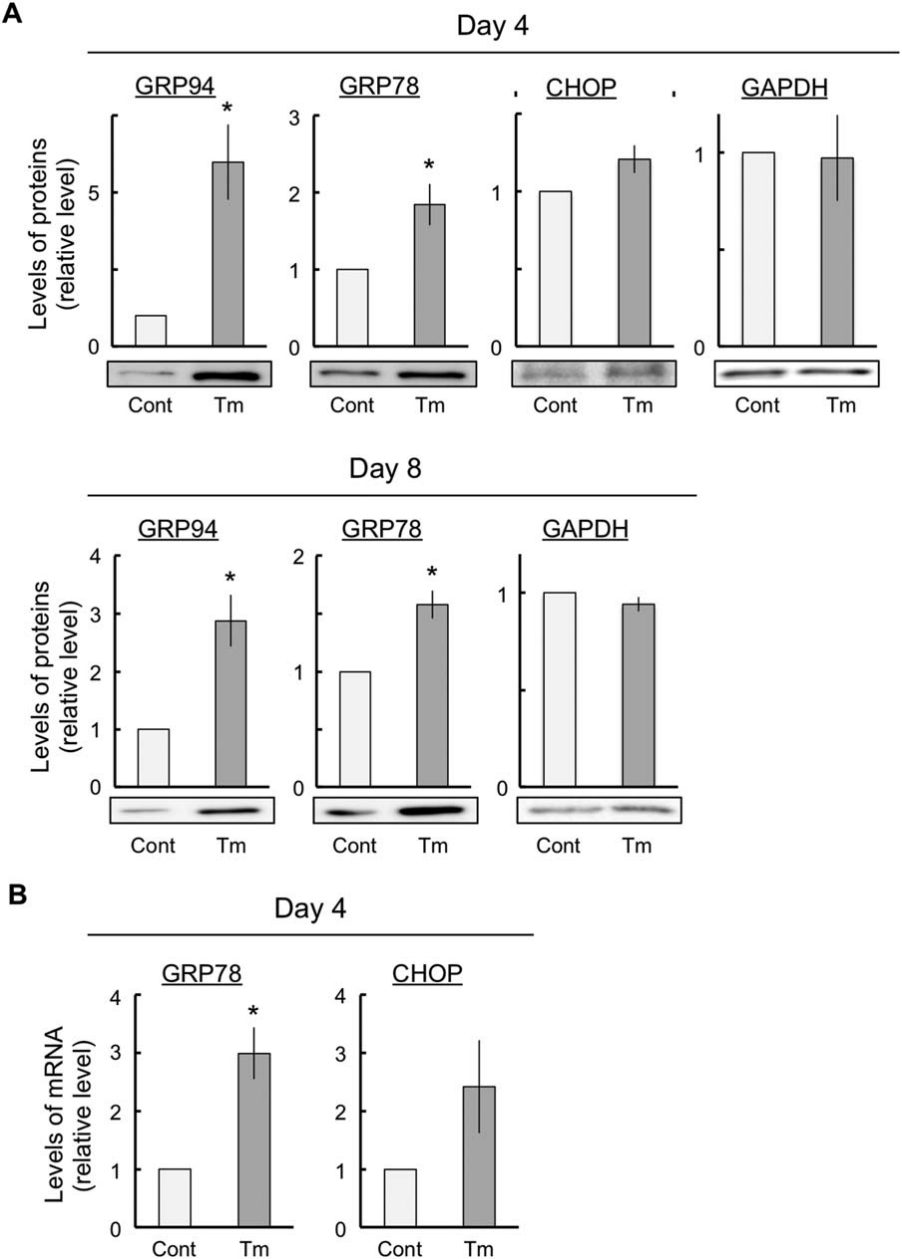
Induction of ER stress with low-concentration tunicamycin. The cells were exposed either to vehicle (control) or to 10 ng/ml tunicamycin for the indicated periods. Total RNA was prepared from the cells and subjected to real-time RT-PCR to determine the expression levels of GRP78 and CHOP. These experiments were conducted at least four times under the same conditions, with similar results. ER stress responses were estimated by immunoblot analyses with anti-KDEL and anti-GADD 153 (CHOP) antibodies (A) and real-time RT-PCR of GRP78 and CHOP mRNA expression (B) for the indicated periods. The expression of GAPDH and β-actin, housekeeping proteins, did not change in these experiments. The values are presented as mean ± SEM from four or five independent experiments. ER, endoplasmic reticulum; GRP, glucose-regulated protein; CHOP, C/EBP homologous protein. *P < 0.05, significantly different from control values.

This study used mouse embryonic carcinoma P19 cells. These cells can quickly differentiate into neurons through NSCs using ATRA. To investigate whether ER stress was induced by 10 ng/ml tunicamycin, we performed an immunoblot assay with the anti-KDEL and anti-GADD 153 (C/EBP homologous protein [CHOP]) antibodies to determine glucose-regulated protein (GRP) 94 and GRP78 expression. GRP94, GRP78, and CHOP are molecular chaperones whose expression levels are known to increase with ER stress. GRP94 and GRP78 expression significantly increased at 4 days (NSCs) and 8 days (neurons) after exposure to tunicamycin (Fig. 2A). CHOP expression showed a tendency to increase at day 4 (NSCs) after exposure to tunicamycin (Fig. 2A). Next, we performed real-time RT-PCR analysis to determine GRP78 and CHOP mRNA expression. GRP78 and CHOP mRNA expression increased at 4 days after exposure to low-concentration tunicamycin (Fig. 2B). Generally, the tunicamycin concentration required to induce ER stress is approximately 100-fold higher than that used in this study. Low-concentration (10 ng/ml) tunicamycin did not induce cell death (data not shown).

### ER Stress Enhanced Differentiation Into Neurons

**Figure 3:**
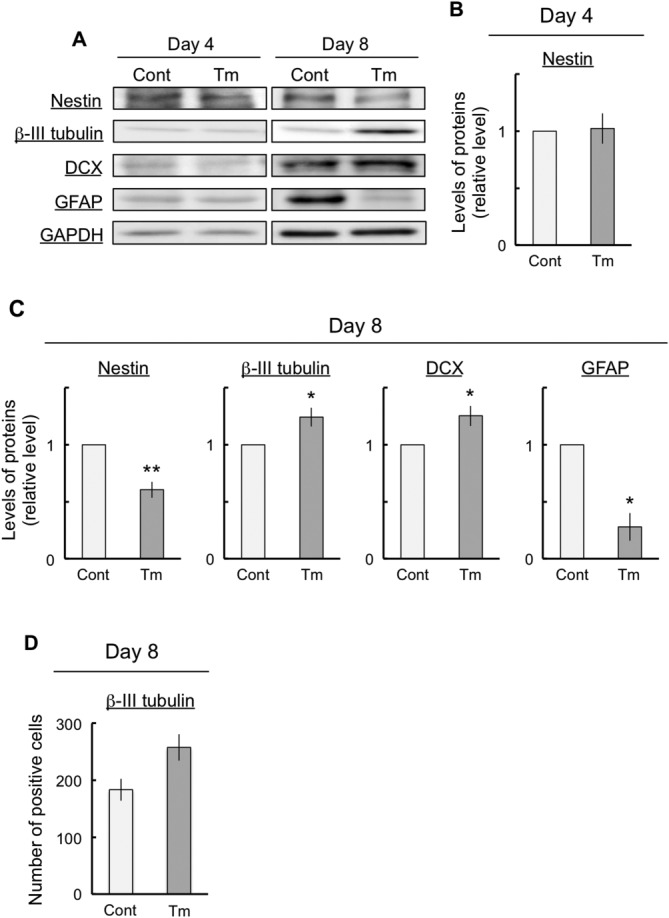
Effects of ER stress on neuronal differentiation. The cells were exposed either to vehicle (control) or to 10 ng/ml tunicamycin for the indicated periods and then subjected to immunoblot analyses to determine the differentiated conditions with various antibodies against marker proteins (A). Quantitative data are presented as levels relative to those for each control (B,C). The number of βIII-tubulin-positive cells was counted in five different randomly selected visual fields (D). The expression of GAPDH, a housekeeping protein, did not change in these experiments. The values are presented as mean ± SEM from four independent experiments. *P < 0.05, **P < 0.01, significantly different from control values.

Tunicamycin induced ER stress without inducing death in P19 cells. These cells differentiated into NSCs and neurons at 4 and 8 days after exposure, respectively. To investigate whether neural differentiation could be influenced by ER stress in P19 cells, we performed an immunoblot assay with antibodies against nestin (an NSC marker), βIII-tubulin (an immature neuronal marker), DCX (an immature neuronal marker), and GFAP (a glial marker) to determine the stage of differentiation in P19 cells. We found that the cells expressed nestin at 4 days with and without tunicamycin, whereas the cells did not express βIII-tubulin, DCX, or GFAP (Fig. 3A). However, at 8 days after exposure, the cells expressed nestin, βIII-tubulin, DCX, and GFAP (Fig. 3A). At 4 days after exposure, tunicamycin had no effect on nestin expression (Fig. 3B). At 8 days after exposure, ER stress led to a decrease in nestin and GFAP expression and to an increase in βIII-tubulin and DCX expression (Fig. 3C). In addition, the number of βIII-tubulin-positive cells showed a tendency to increase at day 8 after induction of ER stress (Fig. 3D). These results indicate that ER stress accelerated neuronal differentiation and inhibited glial differentiation.

### 2-Deoxy-D-Glucose Enhanced Neuronal Differentiation Similarly to Tunicamycin

**Figure 4:**
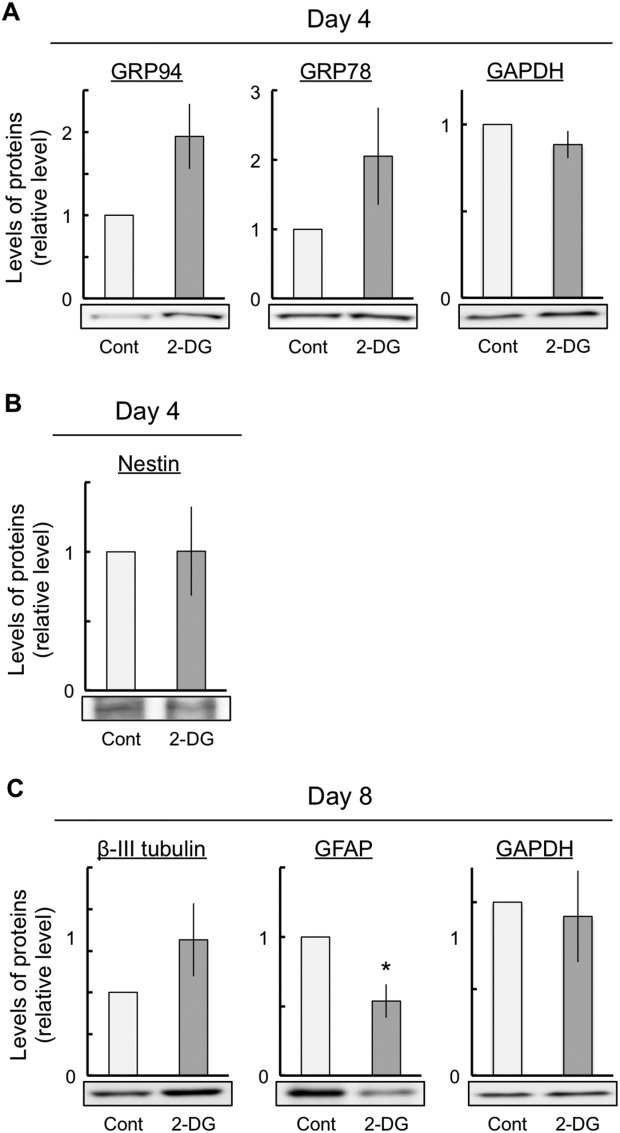
Effects of 2-DG on the ER stress response and the neuronal differentiation. The cells were exposed either to vehicle (control) or to 150 μM 2-DG for the indicated periods and then subjected to immunoblot analyses to determine the differentiated conditions with various antibodies against marker proteins. ER stress responses were estimated by immunoblot analyses with the anti-KDEL antibody (A). Quantitative data of neural marker proteins are presented as levels relative to those for each control (B,C). GAPDH expression did not change in these experiments. The values are presented as mean ± SEM from four or five independent experiments. 2-DG, 2-deoxy-D-glucose. *P < 0.05, significantly different from control values.

To investigate whether another ER stress inducer showed results similar to tunicamycin, we used 150 μM 2-deoxy-D-glucose (2-DG), which is an inducer of ER stress via the inhibition of glycolysis and glycosylation. The expression levels of GRP94 and GRP78 increased at 4 days after exposure to 2-DG (Fig. 4A), whereas the expression levels of nestin were not changed at 4 days after exposure to 2-DG (Fig. 4B). On the other hand, at 8 days after exposure to 2-DG, an increase in the expression levels of βIII-tubulin and a decrease in the expression levels of GFAP were observed (Fig. 4C). These results indicate that ER stress, induced by both 2-DG and tunicamycin, accelerated neuronal differentiation.

### ER Stress Led to Changes in the Expression of the Proneural Genes

**Figure 5:**
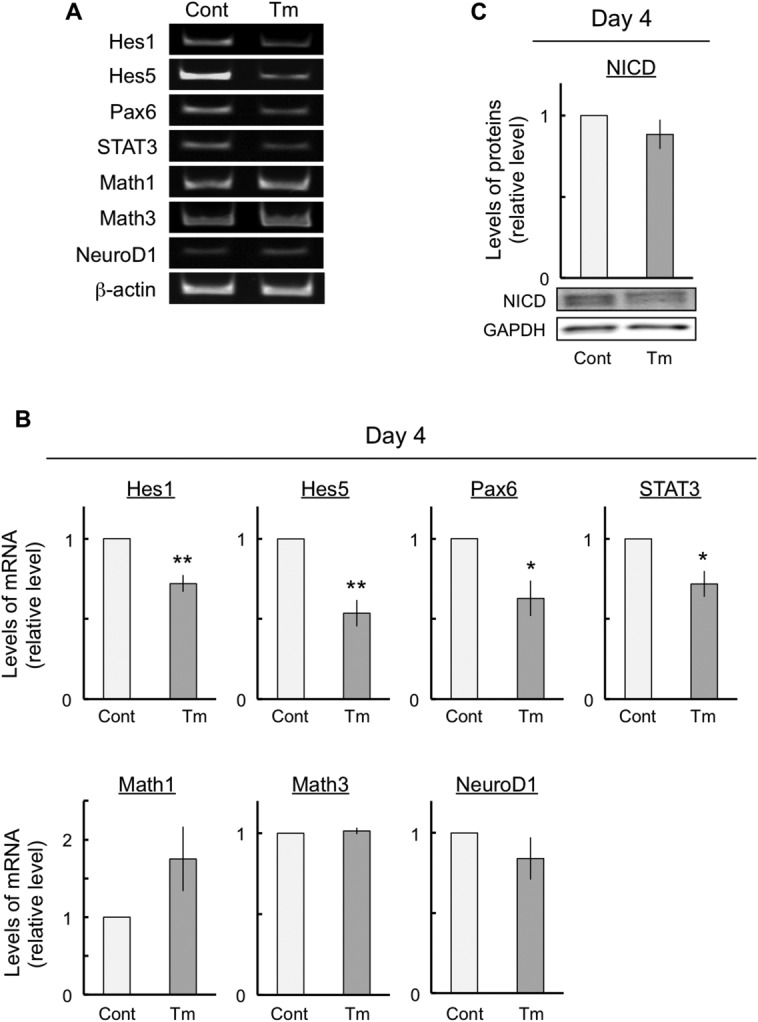
Changes in the expression of proneural genes after ER stress. The cells were exposed either to vehicle (control) or to 10 ng/ml tunicamycin for 4 days. Total RNA was obtained from the cells and subjected to semiquantitative RT-PCR to determine the expression of proneural genes (A). Quantitative data are presented as levels relative to those for each control (B). Protein aliquots were prepared from the cells and subjected to immunoblot analyses to determine the expression levels of the NICD. Quantitative data are presented as levels relative to those for controls (C). The values are mean ± SEM from five independent experiments. ER, endoplasmic reticulum; NICD, Notch1 intracellular domain. *P < 0.05, **P < 0.01, significantly different from control values.

To investigate whether the ER-stress-induced changes in differentiation were related to the expression of proneural genes in P19 cells, we performed a semiquantitative RT-PCR assay designed to measure the expression of proneural genes such as *Hes1*, *Hes5*, *Pax6*, *Stat3*, *Math1*, *Math3*, and *NeuroD1*. The *Hes* family, including *Hes1* and *Hes5*, is involved mainly in glial differentiation. In addition, *Pax6* is involved in NSC suppression, and *Stat3* is involved in the maintenance of undifferentiation, whereas the *Math* family and *NeuroD1* are involved in neuronal differentiation. These proneural genes were expressed at 4 days in both control and tunicamycin-exposed cells (Fig. 5A). We found that Hes1, Hes5, Pax6, and Stat3 mRNA expression significantly decreased with ER stress (Fig. 5A,B). However, ER stress had no effect on Math1, Math3, or NeuroD mRNA expression (Fig. 5A). To investigate whether the ER-stress-induced changes in the basic helix-loop-helix (bHLH) genes were related to Notch signaling in P19 cells, we performed an immunoblot assay to determine NICD expression. NICD converts the transcription factor CBF1/CSL from a gene repressor to a gene activator and regulates the transcription of the *Hes* family. We found that ER stress did not affect NICD expression (Fig. 5C).

### Neuronal Maturation Was Inhibited by ER Stress

**Figure 6:**
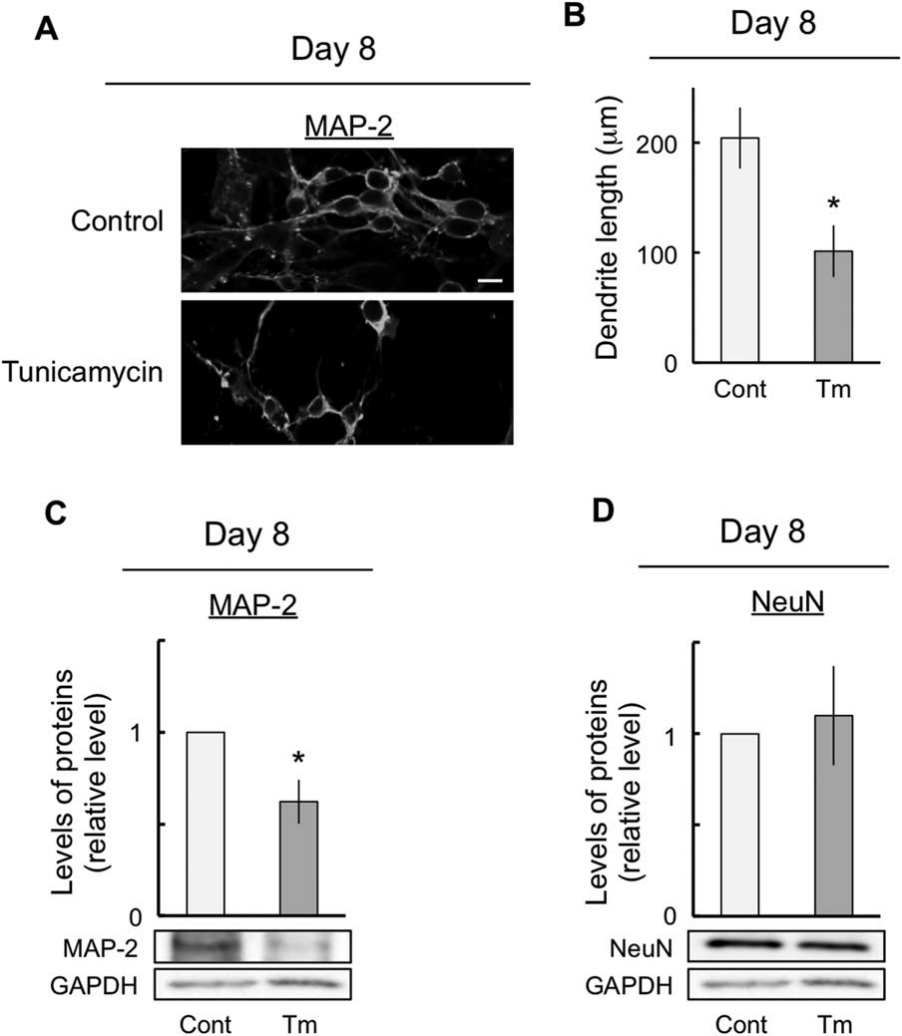
Inhibition of neuronal maturation by ER stress. The cells were exposed either to vehicle (control) or to 10 ng/ml tunicamycin for 8 days. At 8 DIV, differentiated cells were stained with the antibody against MAP-2 (A). B: The dendrite length was measured as described for A. Protein aliquots were prepared from the cells and subjected to immunoblot analyses to determine MAP-2 (C) and NeuN (D) expression. Quantitative data are presented as levels relative to those for controls (C,D). The expression of GAPDH, a housekeeping protein, did not change in these experiments. The values are presented as mean ± SEM from four independent experiments. ER, endoplasmic reticulum; DIV, days in vitro; MAP-2, microtubule-associated protein-2; NeuN, neuronal nuclei protein. *P < 0.05, significantly different from control values. Scale bar = 20 μm.

To investigate whether ER stress was involved in the maturation of neurons in differentiated P19 cells, we performed immunocytochemistry and the immunoblot assays to examine neurite outgrowth. Specifically, at 8 days after exposure, the cells were immunostained with antibodies against dendrite marker MAP-2 in both control and tunicamycin-exposed cells (Fig. 6A). For control cells, we observed that the dendrites of MAP-2-positive cells were long and extended (Fig. 6A). On the other hand, for tunicamycin-exposed cells, we observed that the dendrites of MAP-2-positive cells remained short (Fig. 6B). Cell lysates were detected by immunoblotting with antibodies against MAP-2 in control and tunicamycin-exposed cells at 8 days after exposure (Fig. 6C). The results of the immunoblot assay showed a marked decrease in the expression levels of the dendrite marker MAP-2 in tunicamycin-exposed cells (Fig. 6C). These results were similar to those obtained in the immunocytochemistry assay. In contrast, tunicamycin had no effect on MTT reduction, which depends on the number of surviving cells (data not shown), and NeuN expression, which is also a marker of mature neurons (Fig. 6D). These results indicate that ER stress shortened the length of the dendrites without influencing the survival of mature neurons.

### Deviations in Neuronal Differentiation and Maturation by ER Stress Were Improved by the Suppression of HRD1 Expression

**Figure 7:**
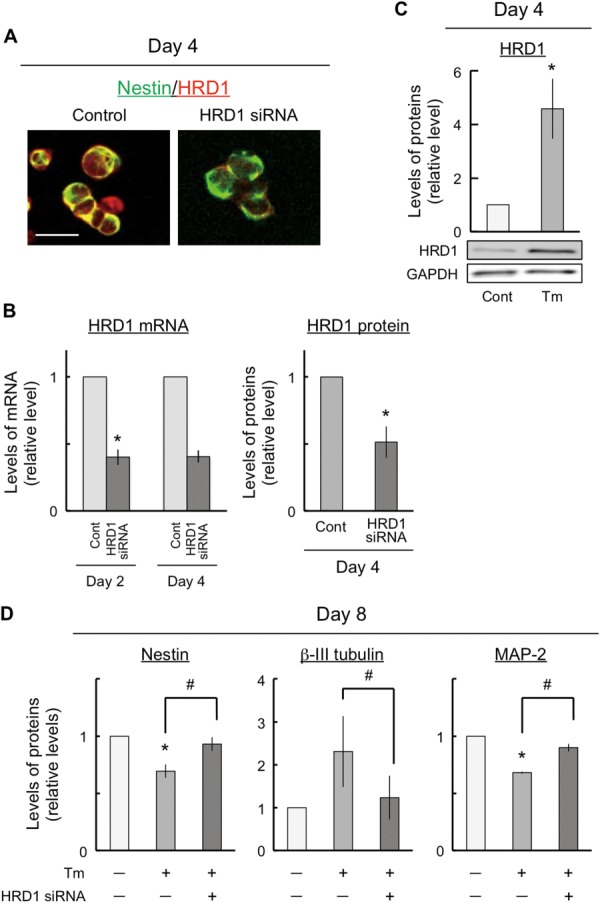
Effects of suppression of ubiquitin ligase HRD1 expression on neuronal differentiation and maturation. The cells were exposed either to control or to 10 ng/ml tunicamycin for 4 or 8 days and HRD1 was transiently suppressed by using HRD1 siRNA following differentiation into NSCs. At 4 days, the cells were harvested and dispersed for replating on dishes that had been previously coated with poly-L-lysine. After incubation in the medium for 1 hr, the cells were fixed for immunostaining with nestin (green) and HRD1 (red; A). Total RNA and protein aliquots were prepared from the cells and subjected to real-time RT-PCR and immunoblot analyses to determine the efficiency of HRD1 knockdown at days 2 and 4. Quantitative data are presented as levels relative to those for controls (B). Protein aliquots were prepared from the cells and subjected to immunoblot analyses to determine the expression levels of HRD1. Quantitative data are presented as levels relative to those for controls (C). The cells were exposed either to control or to 10 ng/ml tunicamycin for 8 days, and HRD1 was transiently suppressed by using HRD1 siRNA following differentiation into NSCs. Quantitative data are presented as levels relative to those for controls (D). The expression of GAPDH, a housekeeping protein, did not change in these experiments. NSC, neural stem cell. *P* < 0.05, significantly different from control values. #*P* < 0.05, significantly different from the values obtained for cells that were treated with tunicamycin alone. Scale bar = 15 μm.

HRD1 is localized in the ER, its expression is induced during ER stress, and it prevents cell death during ER stress (Kaneko et al., 2002; Carvalho et al., 2006). To examine the role of HRD1 in ER stress, we first performed immunocytochemistry to determine the localization of HRD1 in the NSCs differentiated from P19 cells. The cells were immunostained with antibodies against nestin and HRD1 in control cells (Fig. 7A). HRD1 was colocalized in nestin-positive NSCs and was decreased in HRD1-suppressed cells (Fig. 7A). We performed real-time RT-PCR and immunoblot analysis to investigate the efficiency of HRD1 knockdown. We found that HRD1 mRNA expression and protein levels were inhibited to approximately 50% in HRD1-suppressed cells (Fig. 7B). Next, we studied whether HRD1 expression was induced by ER stress. Specifically, we performed immunoblot analysis to investigate HRD1 expression. We found that HRD1 expression was positively induced by ER stress in P19 cells (Fig. 7C). Next, we investigated whether ER-stress-induced neuronal differentiation and stress-induced neurite retraction were mediated by positive HRD1 expression. Subsequently, we examined the effects of the suppression of HRD1 expression using HRD1 siRNA on nestin, βIII-tubulin, and MAP-2 expression. HRD1 mRNA expression was suppressed to less than 50% by siRNA (data not shown). As a result, the changes in nestin, βIII-tubulin, and MAP-2 expression induced by exposure to tunicamycin were diminished by the suppression of HRD1 expression (Fig. 7D).

## Discussion

This study has investigated the effects of ER stress on neuronal differentiation and maturation using P19 cells. We continuously exposed P19 cells to 10 ng/ml tunicamycin for 8 days (Fig. 1). Tunicamycin exposure clearly resulted in an increased protein expression of the ER chaperones GRP94/78 and CHOP both at 4 and 8 days (Fig. 2A). Moreover, the expression levels of GRP78 and CHOP mRNA, which are ER stress markers, were significantly increased at 4 days (Fig. 2B). This increase indicated that ER stress was clearly induced by tunicamycin. However, under these conditions, exposure to tunicamycin did not affect cell viability and proliferation within the first 8 days of exposure (data not shown). Therefore, 10 ng/ml tunicamycin induced ER stress without affecting cell survival under these conditions. Most previous studies regarding tunicamycin-induced ER stress used tunicamycin concentrations that were 100–1,000-fold higher than that used in this study, and it was used for cell death analysis for a shorter term than the exposure duration in this study (Lin et al., 2012). Therefore, to study neuronal differentiation and maturation, we decided to treat cells continuously with 10 ng/ml tunicamycin to obtain a lasting induction of mild ER stress.

In this study, ER stress markedly decreased the NSC marker nestin and the glial marker GFAP expression after the induction of neuronal differentiation (Fig. 3). In addition, ER stress clearly increased the neuronal markers βIII-tubulin and DCX expression after the induction of neuronal differentiation (Fig. 3). The decrease in nestin expression indicated that ER stress enhanced differentiation from NSCs without inducing cell death. In addition, βIII-tubulin and DCX expression and the number of βIII-tubulin-positive cells increased, but GFAP expression decreased. This indicates that ER stress enhanced differentiation into neurons and that ER stress inhibited differentiation into glial cells without inducing cell death. However, this raises the possibility that ER-stress-induced neuronal differentiation was a specific effect of tunicamycin. Therefore, we performed a similar series of experiments in which the cells were exposed to 2-DG. 2-DG led to an increase in ER stress (Fig. 4). In addition, 2-DG increased the expression levels of βIII-tubulin and decreased the expression levels of GFAP (Fig. 4). These results obtained with 2-DG were identical to those obtained with tunicamycin. Therefore, ER stress may induce the enhancement of neuronal differentiation and the inhibition of glial differentiation.

bHLH proneural genes regulate the neuronal differentiation of embryonic cells and NSCs during the developmental process. This set of genes includes repressor-type *Hes1*, *Hes5*, *Pax6*, and *Stat3* and activator-type *Math1*, *Math3*, and *NeuroD1*. *Hes1* and *Hes5* are regulated downstream of Notch signaling, which controls the maintenance of progenitor cells and the timing of their differentiation in various tissues and organs, enhances glial differentiation, and suppresses neuronal differentiation (Apelqvist et al., 1999; Ohtsuka et al., 1999; Cau et al., 2000; Hojo et al., 2000; Hatakeyama et al., 2004; Sumazaki et al., 2004; Fre et al., 2005; Kageyama et al., 2005). Deficits in *Pax6* function inhibit astrocyte maturation and induce the retention of neural stem-like characteristics (Sakurai and Osumi, 2008). *Stat3* is activated by the Notch—Hes signaling pathway, and this activation is essential for the maintenance of radial glial cells and the differentiation of astrocytes (Kamakura et al., 2004). *Math1* is essential for the proper development of cerebellar granule cells (Ben-Arie et al., 1997). *Math3* and *NeuroD* are essential for the genesis of retinal amacrine cells. Coexpression of bHLH and homeobox genes is required for the specification of the correct neuronal subtype (Inoue et al., 2002). We believe that the changes in the expression levels of Hes1, Hes5, Pax6, and STAT3 mRNA may result in the enhancement of neuronal differentiation and in the inhibition of glial differentiation. Notch activation, however, was unaffected by ER stress, although Hes1 and Hes5 mRNA activation downstream of Notch was suppressed by ER stress (Fig. 5C). Therefore, these results may indicate that the enhancement of neuronal differentiation and the inhibition of glial differentiation by ER stress were caused not only by the changes in the expression levels of bHLH proneural genes but also by other mechanisms.

It was apparent that the neurite of MAP-2-positive cells was shortened by ER stress (Fig. 6A). In fact, ER stress led to a decrease of approximately 50% in the dendrite length (Fig. 6B). We found that the expression levels of the dendrite marker MAP-2 were significantly decreased by ER stress, although neuronal differentiation was promoted by this stress (Figs. 3, 6C). Moreover, 2-DG-induced ER stress decreased the expression level of MAP-2 (data not shown). In addition, ER stress decreased MAP-2 expression, although mature neuronal marker NeuN expression (Fig. 6D) and the number of survival cells (data not shown) were unchanged. In brief, the number of mature neurons did not change but the dendrite length decreased with ER stress. Therefore, these results indicated that dendrite outgrowth was possibly inhibited by ER stress. However, in this study, neuronal differentiation was promoted by ER stress (Fig. 3). Thus, neuronal maturation, including dendrite outgrowth, might have been inhibited because of a disruption in the proportion of neuronal differentiation and an enhancement in the aberrations in neuronal differentiation due to ER stress. In fact, Jang et al. (2011) reported that ER stress inhibited osteoblast differentiation by increasing cAMP response element-binding protein H followed by suppressing BMP2-induced old astrocyte specifically induced substance (OASIS) expression in mouse calvarial-derived osteoblasts. In addition, OASIS modulated unfolded protein response signaling and controlled astrocyte differentiation (Saito et al., 2012). Therefore, we suggest that ER stress resulted in aberrant neuronal differentiation that was triggered by the inhibition of neuronal maturation.

It is unclear whether ER stress was involved in the inhibition of neuronal maturation. We reported (Kaneko et al., 2007) that ER stress resulted in an increase in the expression levels of HRD1 (see also Iida et al., 2011). We further hypothesize that ER stress may be involved in the inhibition of neuronal maturation through HRD1. In this study, some defects in the expression of marker proteins such as nestin (NSCs), βIII-tubulin (neurons), and MAP-2 (dendrite) that were caused by ER stress were observed (Figs. 3, 6). All these defects in expression were improved by inhibiting HRD1 expression (Fig. 7). Therefore, these results indicate that the ER-stress-induced defects in neuronal differentiation and neuronal maturation might have been mediated by HRD1. It has been reported that strong ER stress caused defects in cell growth and development (Kitao et al., 2004; Han et al., 2013). In addition, strong ER stress was also involved in cell death (Galehdar et al., 2010). Therefore, this study used mild ER stress, which had no effect on neuronal cell viability. Furthermore, the cells were exposed to mild ER stress for a long period (8 days) during their differentiation from embryonic cells to neurons (Fig. 1). We assumed that this condition of mild ER stress resembles lifestyle diseases such as diabetes. Mild ER stress is induced in diabetes, which involves neural damage (Allen et al., 2004). However, it is unclear how the diabetes-related pathological changes induce neural damage and defects in neuronal maturation processes such as neurite outgrowth. The results of the present study suggest that HRD1 probably participates in the defects associated with ER-stress-induced neuronal maturation. Therefore, we suggest that HRD1 could be involved in defects in neuronal maturation associated with mild ER stress. Hoeck et al. (2010) and Kim et al. (2009) reported that the regulation of neuronal differentiation and neuronal maturation was associated with the control of transcriptional activity through the ubiquitination of histones. The results of this study suggest that the changes in differentiation induced by ER stress may be controlled by the regulation of transcriptional activity, regardless of the functions of HRD1. The expression of bHLH proneural genes, which are involved in neuronal differentiation, was inhibited by ER stress (Fig. 5). However, the inhibition of expression of these genes was not improved via the suppression of HRD1 expression using HRD1 siRNA (data not shown). However, HRD1 may control neuronal maturation during mild ER stress. The ER-stress-induced inhibition of dendrite marker protein (MAP-2) expression was improved by HRD1 suppression (Fig. 7). In brief, the inhibition of HRD1 expression led to an improved neuronal maturation, including neurite outgrowth. Therefore, these results indicate that HRD1 might have a regulatory function in neuronal maturation but not in neuronal differentiation through transcriptional regulation.

In conclusion, we sought to determine whether neurodevelopment, including neuronal maturation and differentiation, was regulated by mild ER stress without inducing neuronal cell death. ER stress resulted in increases in the number of neurons in the cells that differentiated into neurons. However, ER stress resulted in a marked suppression of neurite outgrowth. In contrast, the suppression of HRD1 abolished ER-stress-induced events such as the enhancement of neuronal differentiation. These results indicate that ER stress may promote neuronal differentiation from NSCs but inhibit neurite outgrowth. These events might be associated with an increase in HRD1 expression. Therefore, elucidation of the mechanisms underlying the ER-induced changes in neuronal development may provide a novel pathogenic mechanism for developmental disorders such as autism-spectrum disorders.
